# Residual Breast Sarcoma Left to Heal by Secondary Intention Following Wide Local Excision: An Unorthodox Approach to a Rare Malignancy

**DOI:** 10.7759/cureus.10433

**Published:** 2020-09-13

**Authors:** Talal Almas, Salman Hussain, Maryam Ehtesham, Muneeb Ullah, Muhammad Kashif Khan

**Affiliations:** 1 Internal Medicine, Royal College of Surgeons in Ireland, Dublin, IRL; 2 General Surgery, Maroof International Hospital, Islamabad, PAK; 3 Surgical Oncology, Federal Government Poly Clinic (Post Graduate Medical Institute), Islamabad, PAK; 4 Surgical Oncology, Maroof International Hospital, Islamabad, PAK

**Keywords:** breast sarcoma, phyllodes tumour, secondary intention

## Abstract

Breast sarcomas remain a rare malignancy and are noted to portend a particularly dismal prognosis. Due to their high rates of recurrence, a surgical excision with negative margins remains the preferred treatment modality. Nevertheless, their sparse prevalence often poses a diagnostic conundrum. In this report, we chronicle the case of a 46-year-old female with a recurrent breast sarcoma that was treated with wide local excision. Interestingly, no flap reconstruction, skin graft surgery, or primary wound closure was performed, and the resultant wound was left to heal by secondary intention. The patient continues to do well to date, with no postoperative complications.

## Introduction

Breast sarcomas, which arise from the mesenchymal elements of the breast, are a rare malignancy that are noted to manifest a remarkable heterogeneity. They account for less than 1% of all breast malignancies and less than 5% of all sarcomas [1]. Breast sarcomas are categorized as either primary pure breast sarcomas or breast sarcomas secondary to radiotherapy, with the former being much rarer due to scarce data and very low prevalence. Breast sarcomas are usually diagnosed in the fifth and sixth decades of life and occur almost exclusively in females. Their typical presentation includes a rapidly growing, well-defined mass in the breast. In rare instances, breast sarcomas can be associated with overlying skin changes or pain [2]. The development of breast sarcoma is associated with various genetic conditions, including Li-Fraumeni syndrome and neurofibromatosis type 1. Pertinently, a strong association between the development of breast sarcomas and TP53 mutations has been established [3]. Breast sarcomas encompass a diverse array of histologic subtypes, including fibrohistiocytoma, fibrosarcoma, angiosarcoma, and spindle cell sarcoma; however, malignant phyllodes tumors are considered a distinct entity owing to the presence of epithelial components [2]. Phyllodes tumor of the breast, previously known as cystosarcoma phyllodes, is a rare fibroepithelial neoplasm of the breast and constitutes only 0.3-0.5% of all breast tumors [4,5]. Since both breast sarcomas and malignant phyllodes tumors present with an exceedingly identical constellation of symptoms, their prompt diagnosis remains a conundrum. In our case, a female patient presented with a tumor that had been initially misdiagnosed and thus managed as a malignant phyllodes tumor. Upon recurrence of the tumor, a final diagnosis of spindle cell sarcoma, a rare histologic subtype of breast sarcoma, was confirmed on histopathology report. Due to the patient’s preferences, a latissimus dorsi (LD) myocutaneous flap reconstruction surgery was not performed. The wound was kept on daily dressings for a period of four months and was allowed to heal by secondary intention.

## Case presentation

We discuss the case of a 46-year-old female who presented with a history of a recurrent lump in her right breast with no known comorbid conditions. Three years prior to the presentation, the patient had been examined at another healthcare facility for the presence of a tumor in the right breast. The physicians at this facility had performed breast conservation surgery to excise the tumor. However, the patient had experienced a subsequent recurrence. A core biopsy had been then performed, but the histopathology had turned out negative for an underlying malignancy. A modified radical mastectomy (MRM) had been then performed for her recurrent tumor, divulging positive margins on biopsy.

**Figure 1:**
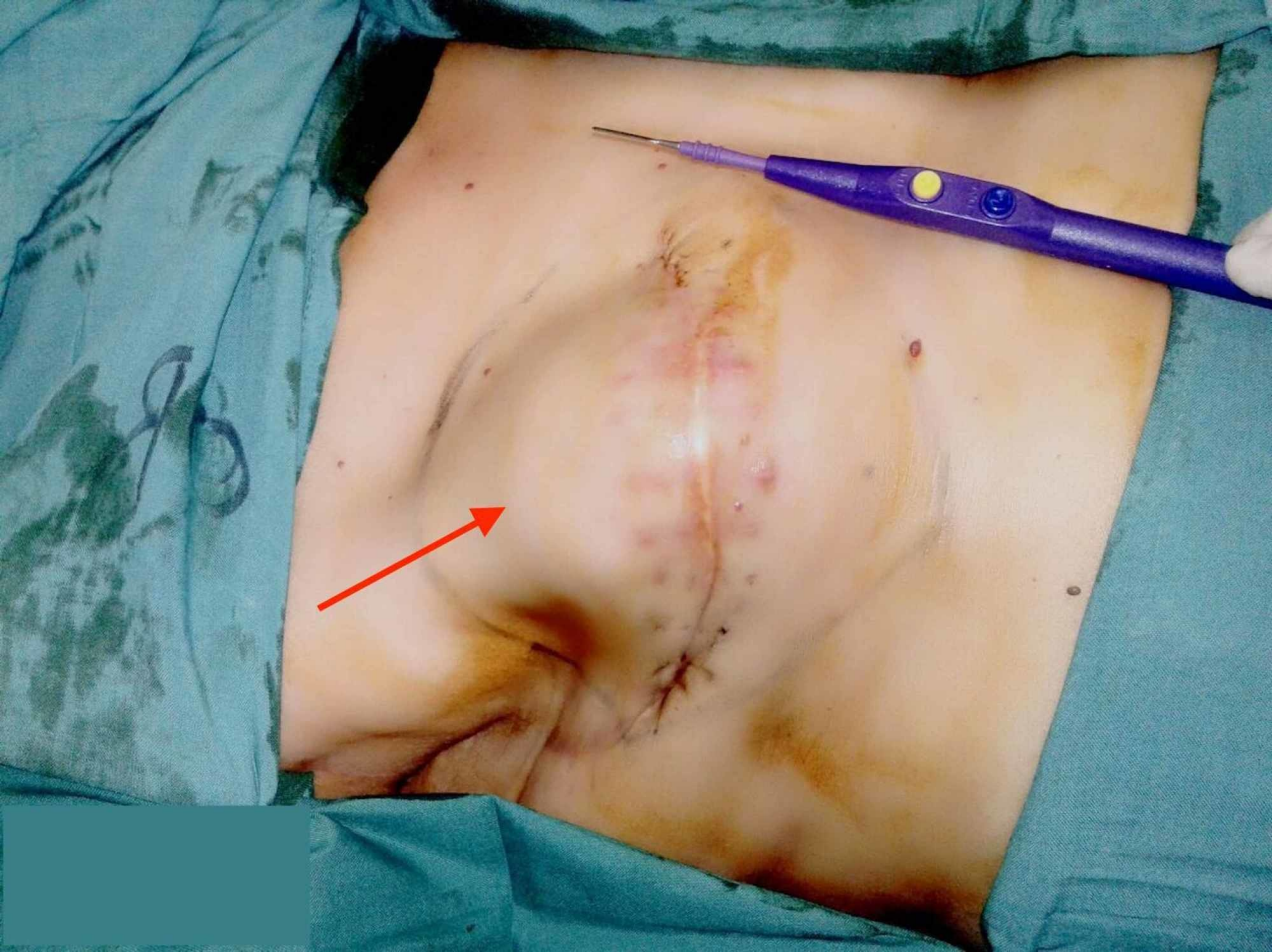
Preoperative image The image shows a residual lump in the right breast (red arrow), measuring 12 x 10 cm in size, fixed to underlying pectoralis major muscle with limited right arm abduction

Thereafter, the patient presented to us with a residual mass in the right breast (Figure 1).

A core needle biopsy of the mass was subsequently performed; the histopathology report revealed a herringbone and short fascicular pattern with the cells appearing to be spindle-shaped with eosinophilic cytoplasm demonstrating vesicular to hyperchromatic nuclei and the presence of inconspicuous nucleoli. The immunohistochemical analysis further divulged the absence of cytokeratin markers and p63 and the presence of desmin. These findings are delineated in Table 1.

**Table 1:** The status of various immunohistochemical stains CD: cluster of differentiation; CK: cytokeratin; EMA: epithelial membrane antigen

Immunohistochemical stain	Status
Cytokeratin	Negative
CD34	Negative (highlights blood vessels)
P63	Negative
Cam 5.2	Negative
Desmin	Positive (highlights skeletal muscle)
S100	Negative
334bE12	Negative
CK5/6	Negative
EMA	Negative

**Figure 2:**
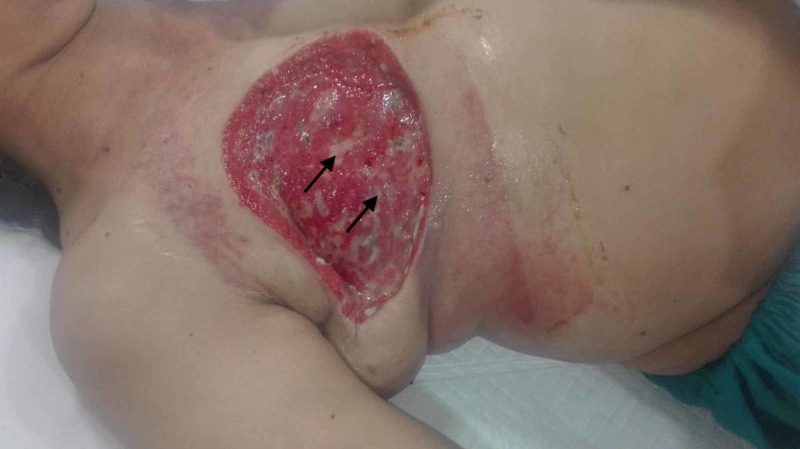
Postoperative image The image shows wound healing by secondary intention as evidenced by the granulation tissue at the base (black arrows)

These immunohistochemical findings insinuated a final diagnosis of a spindle cell sarcoma. Owing to the extensive scarring from the previously performed MRM, the feasible option for treatment was a wide local excision along with an LD flap reconstruction surgery. The flap reconstruction surgery was planned in order to cover up the large wound postoperatively. However, the patient refused the surgery, citing her financial constraints. Instead, a wide local excision with a negative margin resection was performed, allowing the excision of the major/minor pectoralis muscle and exposing the ribs beneath. Interestingly, no flap construction surgery was performed. A postoperative image on the sixth day of the surgery revealed the presence of granulation tissue at the base, facilitating the healing of the wound by secondary intention despite a surgical operation with deep margins (Figure 2).

The patient continues to do well to date, with no recurrence or complications.

## Discussion

Primary sarcomas of the breast are exceedingly uncommon and account for only 0.2-1% of all breast malignancies [6]. The reported five-year survival rate ranges from 40-91% [1,7,8]. The optimal management for primary breast sarcomas remains a conundrum for surgeons and depends on a multitude of factors including histologic subtype, the grade of the tumor, size of the tumor, and the advancement of the disease [6]. Nevertheless, surgical resection remains the mainstay of treatment for primary breast sarcomas, and complete microscopic resection of the primary tumor remains pivotal in determining the disease-free survival (DFS), overall survival (OS), and local control of the disease [7,9]. Pandey et al. have reported that the most important prognosticator is the margin of surgical resection. The three-year survival rate was 0% in patients with positive margins and 33% in patients with negative margins (p=0.05) [9]. Similarly, Blanchard et al. have reported that wide local excision yielded equally good local control as compared to mastectomy as long as complete removal of the tumor was achieved [10].

In our case, the initial histopathological report indicated no cytokeratin markers except for desmin and, as a result, the tumor was initially misdiagnosed as malignant phyllodes for which a wide local excision was performed. Three months later, the tumor recurred, and an MRM was thus performed. However, upon the third recurrence of the tumor, a core needle biopsy was performed prior to surgery to analyze the lesion. Further investigations revealed that neurofibromatosis was present in the dermal layer of the skin with an underlying spindle cell sarcoma showing no lymph node metastasis, positive margin resection, and angiosarcomatous elements in the histopathological analysis. Due to the extensive involvement of the deep tissue, a wide local excision was performed to ensure completeness of resection.

There have been dramatic advancements in the field of reconstructive surgery following the resection of breast tumors. Even so, the uptake of reconstructive surgery following resection of breast sarcomas, particularly due to the difficulty of management in cases of local recurrence, remains enigmatic. Current literature suggests similar prognostic outcomes in patients undergoing reconstructive surgery in comparison to patients who do not [11]. Therefore, patients are routinely offered and advised to undergo reconstructive operations to preserve the quality of life immediately following tumor resection. Our patient was offered an LD flap reconstruction following MRM but refused surgery due to financial issues. Our case, therefore, delineates an unorthodox approach of allowing the wound to heal by secondary intention. Another treatment option was flap reconstruction surgery, but the patient did not give consent for it due to which we chose this method for wound healing. The standard, conventional management of surgical wounds left to heal by secondary intention involves daily or more frequent dressing changes, occasionally with the packing of the wound cavity [12]. Our patient had undergone a wide local excision with removal of the underlying pectoralis major muscle. On the sixth day postoperatively, granulation tissue was seen at the wound base, indicating healing of the wound by secondary intention. Thereafter, the wound was kept on daily dressing changes for a period of four months. The patient continues to thrive, with no recurrence or postoperative complications. The disadvantages of our approach include prolonged hospital stay and increased risk of infection. Nevertheless, the latter can be tackled by appropriate prophylactic antimicrobial regimen and good postoperative care.

## Conclusions

Breast sarcomas are a rare subset of all breast malignancies and are associated with high rates of recurrence and subsequent mortality. Wide local excision is considered to be the optimal surgical modality for breast sarcomas. In cases of deep excisions, an LD flap reconstruction surgery is traditionally performed to cover the resultant wound. However, in rare instances such as ours, such wounds are left to heal by secondary intention, which is an unorthodox yet cost-effective treatment modality in developing countries.

## References

[1] Pollard SG, Marks PV, Temple LN, Thompson HH (1990). Breast sarcoma. A clinicopathologic review of 25 cases. Cancer.

[2] Lim SZ, Ong KW, Tan BK, Selvarajan S, Tan PH (2016). Sarcoma of the breast: an update on a rare entity. J Clin Pathol.

[3] Birch JM, Alston RD, McNally RJ (2001). Relative frequency and morphology of cancers in carriers of germline TP53 mutations. Oncogene.

[4] Anand P, Sarin N, Butti AK, Singh S (2018). Malignant phyllodes tumor in a young female: a case report. Gulf J Oncol.

[5] Rowell MD, Perry RR, Hsiu JG, Barranco SC (1993). Phyllodes tumors. Am J Surg.

[6] Cil T, Altintas A, Pasa S, Buyukbayram H, Isikdogan A (2008). Primary spindle cell sarcoma of the breast. Breast Care (Basel).

[7] Adem C, Reynolds C, Ingle JN, Nascimento AG (2004). Primary breast sarcoma: clinicopathologic series from the Mayo Clinic and review of the literature. Br J Cancer.

[8] Callery CD, Rosen PP, Kinne DW (1985). Sarcoma of the breast. A study of 32 patients with reappraisal of classification and therapy. Ann Surg.

[9] Pandey M, Mathew A, Abraham EK, Rajan B (2004). Primary sarcoma of the breast. J Surg Oncol.

[10] Blanchard DK, Reynolds CA, Grant CS, Donohue JH (2003). Primary nonphylloides breast sarcomas. Am J Surg.

[11] Toesca A, Spitaleri G, De Pas T (2012). Sarcoma of the breast: outcome and reconstructive options. Clin Breast Cancer.

[12] Chetter IC, Oswald AV, McGinnis E (2019). Patients with surgical wounds healing by secondary intention: a prospective, cohort study. Int J Nurs Stud.

